# Bathing-related accidents requiring ambulance dispatches in relation to age and ambient temperature in Nagoya, Japan: differences between detached houses and apartment buildings

**DOI:** 10.1265/ehpm.25-00123

**Published:** 2025-09-17

**Authors:** Akihiko Narisada, Tomohiro Umemura, Nauta Yamanaka, Kohta Suzuki

**Affiliations:** 1 School of Nursing & Health, Aichi Prefectural University, Nagoya, Aichi 463-8502, Japan; 2 School of Health and Sport Science, Chukyo University, Toyota, Aichi 470-0393, Japan; 3 Department of Health and Psychosocial Medicine, Aichi Medical University School of Medicine, Nagakute, Aichi 480-1195, Japan

**Keywords:** Bathing-related accidents, Bathing-related deaths, Japan, Housing type, Aging society, Low ambient temperature, Population-attributable fraction, Age-standardized incidence rate, Time-stratified case-crossover design, Distributed lag nonlinear modeling

## Abstract

**Background:**

Previous studies have shown that old age and cold temperatures are risk factors for bathing-related accidents (BRAs) in Japan. The differences between outdoor and indoor temperatures are believed to depend on the housing type (detached houses or apartment buildings). This study aimed to investigate the associations between age, temperature, and BRAs according to housing type in Japan.

**Methods:**

We included cases in which patients were transported by ambulance from domestic bathrooms between April 2016 and March 2022 in Nagoya city. Age-specific BRA incidence rates measured by 5-year age groups, temperature-specific age-adjusted standardized incidence rates (SIRs) for BRA calculated by temperature quintile groups, and the BRA risk regarding temperature based on a time-stratified case-crossover (CCO) design were compared between detached houses and apartment buildings.

**Results:**

We observed 4,848 ambulance dispatches owing to BRAs (3,083 in detached houses and 1,765 in apartment buildings; SIR for detached houses compared to apartment buildings: 1.37; 95% confidence interval [CI]: 1.33–1.43). The ratio of detached houses to apartment buildings in the age-specific BRA incidence was almost the same in middle-aged people, but it significantly increased from the age of 70 years onward (incidence rate ratio for the 70–74-years age group: 1.74; 95% CI: 1.43–2.11). Temperature-specific SIR for detached houses compared to apartment buildings was not significantly different in the hottest temperature quintile but increased significantly in the other colder temperature quintiles (SIR in coldest quintile: 1.56; 95% CI: 1.47–1.66). BRA risk based on CCO design increased significantly with a decrease in temperature in detached houses (risk ratio [RR] for 3 °C: 1.25; 95% CI: 1.05–1.47), but not in apartment buildings (RR for 3 °C: 1.07; 95% CI: 0.86–1.34).

**Conclusions:**

Detached houses had higher BRA incidence rates than apartments. Older age and lower temperatures, which are risk factors for BRAs, were more prevalent in detached houses than in apartment buildings. Thus, public health measures that focus on detached houses are necessary for preventing BRAs in Japan.

**Supplementary information:**

The online version contains supplementary material available at https://doi.org/10.1265/ehpm.25-00123.

## Graphical Abstract

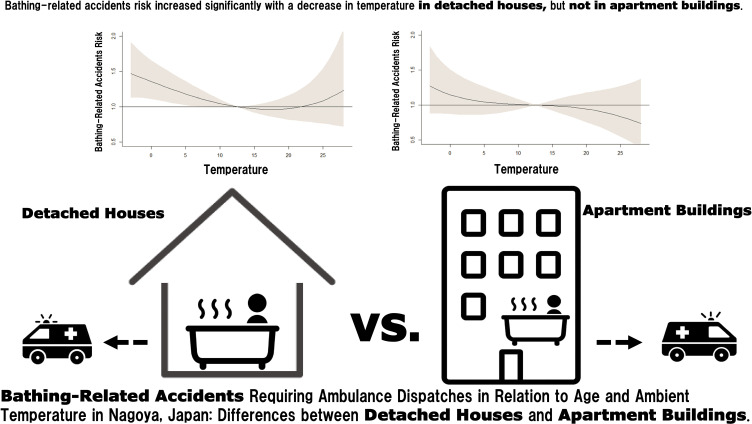

## Introduction

Bathing-related accidents (BRAs), particularly deaths, are a serious public health problem in Japan. Bathing-related deaths are estimated to account for >10% of all sudden deaths, and among older adults, it is almost twice as common as traffic accident deaths [1–4]. Most cases occurred in winter, on days with low temperature, and the victims were mostly individuals aged ≥65 years [2, 3]. Analyzing nonfatal BRAs, which plays a crucial role for preventing bathing-related deaths [5], indicated that those are also associated with old age and low temperatures [5–9].

The high number of BRAs In Japan is thought to be due to the cold bathrooms and dressing rooms in Japanese houses, in addition to the practice of soaking in the bathtub during bathing [2–4]. In Japan, detached houses are less well-insulated and often built of wood, so they are affected by the external temperature in winter and have lower room temperatures than apartment buildings [10–12]. Additionally, in most Japanese detached houses, the changing rooms and bathrooms are located on the north side and have poorly insulated windows [2, 13]. Therefore, BRAs due to low temperatures may vary depending on the housing type, that is, detached houses or apartment buildings. However, this hypothesis is not well-understood.

Thus, this study aimed to investigate whether there are differences in the occurrence of BRAs depending on the housing type (detached houses or apartment buildings) in Japan. In particular, we examined whether the association of BRAs with age and temperature, which are well-known risks [2–4], differs depending on the housing type. As emergency transport is widely available to all residents in Japan [14], we used emergency transport data on BRAs to assess the relationship between age, temperature, and BRAs by housing type.

## Methods

### Study setting

This study used data from Nagoya, a large city in the Chubu region of Japan. The city has an area of 326.46 km^2^, an average temperature of 17.5 °C, and a population of 2.33 million according to the 2020 Population Census [15, 16]. The study protocol was reviewed and approved by the Ethics Committee of the School of Medicine, Aichi Medical University (2022-016).

### Study population

We used data on emergency ambulance dispatches from April 1, 2016, to March 31, 2022, provided by the Nagoya City Fire Department Emergency Services Division, which includes caller demographic information, time and date of incident, location of incident, ambulance activity record, and diagnosis [17, 18]. The incident locations are broadly categorized into residences, public places, workplaces, roads, and others, and the residence information includes the type of residence (detached house, apartment building) and information on the location of occurrence within the residence (living room, stairs, hallway, bathroom, kitchen, toilet, rooftop, elevator, garden, and others) [17]. Diagnoses are broadly categorized as disasters, accidents, self-harm or harm to others, injuries, and acute illness, and illness diagnoses were made by doctors at the time of the first consultation based on the International Classification of Diseases, 10^th^ revision [18]. In Japan, emergency medical services are provided free of charge by local governmental fire defense headquarters, and citizens can call ambulances via the emergency number “119” [14]. We also used the 2020 Population Census as population data for Nagoya City in this study [16].

### Outcome

Using the emergency data, we identified BRA occurrences. We defined a BRA as any ambulance dispatch (for any cause, including injuries or illness, regardless of diagnosis) that occurred in the bathroom of a residence (detached house or apartment building).

### Assessment of housing type

The occurrence of BRAs in detached houses or apartment buildings was assessed based on the location information in the emergency data. Based on the “location code” in the emergency data, BRAs in detached houses or apartment buildings were defined as cases registered as “detached house, bathroom (CODE: 0103)” or “apartment building, bathroom (CODE: 0203),” respectively. While the housing type at the incident location in the emergency data is categorized as detached houses and apartment buildings, the housing type population in the 2020 Census data used to calculate the BRA incidence rate is categorized as detached houses, apartment buildings, and tenement houses. Based on the facts that an apartment building is defined as having multiple households sharing stairs and hallways [19] and that the city government has pointed out that distinguishing between tenement houses and detached houses is difficult [20], the population of tenement houses was considered to be the detached house population in this study, that is, the detached house population was defined as the detached house population plus the tenement house population.

### Environmental data

Meteorological data on the daily minimum ambient temperature and relative humidity in Nagoya City were provided by the Japan Meteorological Agency. Daily minimum ambient temperature and relative humidity were calculated using hourly measurements [21].

### Statistical analysis

For comparisons of characteristics between detached houses and apartment buildings within the BRA cases, comparisons of proportions were made using chi-squared tests and comparisons of continuous variables were made using Student’s t tests. Incidence rates were calculated and compared using population data from the 2020 Census for each residential residence and observation period. Age-adjusted standardized incidence rates (SIRs) were used to compare BRAs between detached houses and apartment buildings. SIRs and 95% confidence intervals (CIs) for BRAs in detached houses were determined from the number of observed cases relative to the number of expected cases, calculated by the age-adjusted incidence rates for BRAs in apartment buildings. The population-attributable fraction (PAF) of people living in detached houses for the risk of BRAs was calculated using Miettinen’s formula [22].

To examine the combined effect of age and housing type, the age-specific incidence rates of BRAs by 5-year age groups during the study period were calculated, stratified by housing type, and compared between both groups. To examine the effect of temperature, the temperature-specific incidence rates of BRAs by daily minimum temperature quintiles (−3.9 to 3.8 °C, 3.9–9.4 °C, 9.5–16.3 °C, 16.3–22.2 °C, and 22.3–28.8 °C) were calculated stratified by housing type. Subsequently, the temperature-specific SIRs for each temperature quintile were also calculated to compare detached houses and apartment buildings.

We further used a time-stratified case-crossover (CCO) design to examine the association between daily minimum temperature and BRAs by housing type, controlling for potential confounding by known and unknown individual- and community-level covariates that do not vary from day to day (e.g., age, sex, socioeconomic status, population density, and behavioral risk factors, such as smoking) [23]. In this design, we defined a “case” day as the day of transport for a BRA and selected three or four “control” days from the corresponding day of week in the same month and year as the “case” day [24]. We estimated risk ratios (RR) and 95% CIs of BRAs associated with daily minimum temperature, using conditional Poisson regression model to compare the minimum temperature on a “case” day (exposure) with that on a “control” day when the person did not become a case [22]. To describe nonlinear association temperature and accident as an exposure–response curve, we applied a distributed lag nonlinear modeling (DLNM) framework [25]. Specifically, a cross-basis function with a natural cubic B-spline basis having a degree of freedom (df) of 3 was used for the temperature, and a natural cubic B-spline basis with df 3 was introduced for the lag. The lag was extended up to 5 days prior, based on previous studies [26, 27]. The choices for the df were based on the model selection criteria, the Quasi Akaike Information Criterion. In the conditional Poisson regression model using this cross-basis function, we additionally adjusted for relative humidity and federal holidays, and 12.5 °C, the midpoint between the highest and lowest daily minimum temperatures during the study period, was used as the reference for calculating RRs.

Furthermore, a subgroup analysis was performed considering age, stratified by the age of 65 years [2, 28]. Subgroup analyses were also performed considering the cause of BRAs and analyses were performed separately for BRAs caused by injury.

Sensitivity analyses were performed to assess the robustness of the findings. First, we calculated the SIRs by excluding the tenement house population from the detached house population. Second, we performed an analysis using DLNM with a lag time of 21 days instead of 5 days. Third, to assess whether our results were robust to the choice of exposure metric, we repeated the DLNM analyses using exposure based on the daily mean temperature rather than the daily minimum temperature. Finally, we performed analyses by excluding cases that occurred during the coronavirus disease 2019 (COVID-19) pandemic [29].

## Results

According to the 2020 Census [16], 997,235 people lived in detached houses (including 27,926 who lived in tenement houses), while 1,262,551 lived in apartment buildings in Nagoya City; a comparison by 5-year age group showed that >10,000 people in the 0–4- and 20–54-years age groups lived in apartment buildings, while more people in each age group over 70 years lived in detached houses (Fig. S1). In other words, residents of detached homes were older.

During the study period, we observed 4,848 ambulance dispatches owing to BRA: 3,083 in detached houses and 1,765 in apartment buildings. The overall incidence rate during the study period was 35.7 per 100,000 person-years. BRA cases occurred on 1,957 of the 2,191 days during the study period (zero cases on 234 days). The most frequent occurrence was 2 cases per day (441 days), and the maximum occurrence per day was 13 cases (three days). Table 1 shows the statistical characteristics of BRAs by housing type. The incidence of BRAs was 51.5 per 100,000 person-years in detached houses and 23.3 in apartment buildings. The SIR for detached houses compared to apartment buildings was 1.37 (95% CI: 1.33–1.43). We calculated the PAF of living in detached houses for the risk of BRAs, and estimated that 14.4% of BRAs could be attributed to detached houses. Among the individuals who were transported owing to BRA, the proportion of men was not significantly different between both housing types, but the mean age and the proportion of individuals aged ≥65 years was higher in those transported from detached houses. The proportions of drowning and CPA cases were higher in detached houses, whereas the proportion of injuries was higher in apartment buildings.

**Table 1:** Characteristics of ambulance dispatch for bathing-related accidents by housing type

**Variables**	**Detached houses**	**Apartment buildings**	***p***
**Residents (person-years)^a^**	5,983,410	7,575,306	
Case			
**Number**	3,083	1,765	
**Mean age (years)**	74.4 (20.4)	61.3 (26.7)	<0.001
**Aged ≥65 years old**	2,602 (84.4%)	1,075 (60.9%)	<0.001
**Men**	1,746 (56.6%)	982 (55.6%)	0.5
**Drowning**	109 (3.5%)	37 (2.1%)	<0.001
**Injury**	401 (13.0%)	319 (18.1%)	<0.001
**CPA**	690 (22.4%)	264 (15.0%)	<0.001
**Weekday onset**	2,032 (65.9%)	1,194 (67.6%)	0.2

**Incident rate (per 100,000 person-years)**	51.5	23.3	<0.001
**SIR (95% CI)**	1.37 (1.33–1.43)	1 (Reference)	

CPA cardiopulmonary arrest; CI confidence interval; SIR age-standardized incidence rate

a: Calculated based on the Japanese 2020 census.

**Fig. 1:**
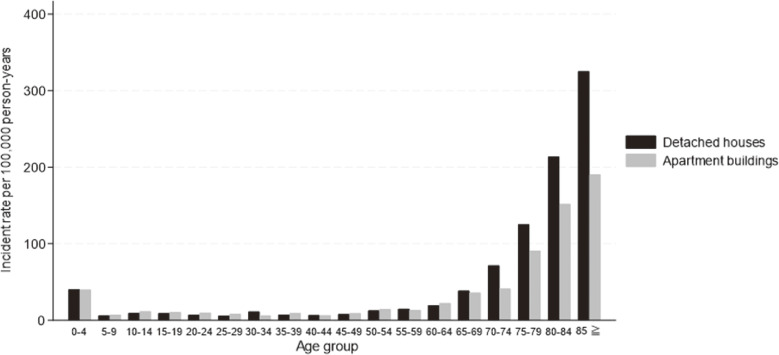
Age-specific incident rate of BRA between detached houses and apartment buildings. Abbreviation: BRA, bathing-related accidents

Figure 1 and Table S1 show the age-specific incidence rates of BRAs by 5-year age groups for detached houses and apartment buildings. The incidence rate of BRAs increased with age in both housing types except for infancy [30] (Fig. 1). The incidence rate ratio of BRAs in detached houses compared to apartment buildings was almost the same for 35–69-years age groups, but increased clearly from the age of 70 years (incidence rate ratio for 70–74-years age group: 1.74; 95% CI: 1.43–2.11) (Table S1).

**Fig. 2:**
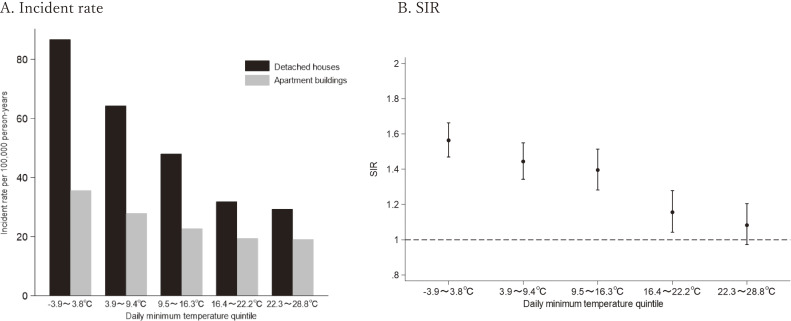
Temperature-specific BRA between detached houses and apartment buildings. 2A. The incidence rates of BRA by daily minimum temperature quintiles. 2B. The SIR for detached houses compared to apartment buildings by daily minimum temperature quintiles. Abbreviation: BRA, bathing-related accidents; SIR, age-standardized incident rate

Figure 2 shows the temperature-specific incidence rates of BRAs stratified by the daily minimum temperature quintiles in detached houses and apartment buildings. The incidence rate of BRAs increased as the temperature reduced in both housing types (Fig. 2A). The SIR for detached houses compared to apartment buildings by temperature quintile was not significantly different in the hottest class (SIR in the 22.3–28.8 °C quintile: 1.08; 95% CI: 0.97–1.20), but it increased significantly in the other colder temperature quintiles (SIR in the −3.9 to 3.8 °C quintile: 1.56; 95% CI 1.47–1.66) (Fig. 2B).

**Fig. 3:**
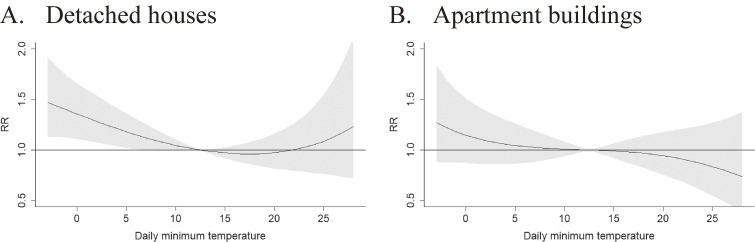
Cumulative exposure-response curve of the association between daily minimum temperatures and BRA. 3A. Detached houses. 3B. Apartment buildings. Abbreviation: BRA, bathing-related accidents; RR, risk ratio

Figures 3 and S2A and Table S2 show the nonlinear relationship between daily minimum temperature and BRAs on the day (lag 0) overall (Fig. S2A) and by housing type (Fig. 3) using the DLNM framework. In the analysis of all houses (detached houses and apartment buildings), we found a monotonic association between the daily minimum temperature and the relative risk of BRAs on that day; however, there was no clear evidence of a threshold (Fig. S2A and Table S2). Figure S2B shows three-dimensional graphs of the exposure-response relationship between daily minimum temperature, lag (0–5 days), and BRAs. In detached houses, the risk of BRAs significantly increased with a decrease in the daily minimum temperature. However, in apartment buildings, a decrease in the temperature did not significantly increase the risk of BRAs (Fig. 3). For example, the RRs of ambulance dispatches on the same day (lag 0) at 3 °C for detached houses and apartment buildings were 1.25 (95% CI: 1.05–1.47) and 1.07 (95% CI: 0.86–1.34), respectively (Table S2).

**Fig. 4:**
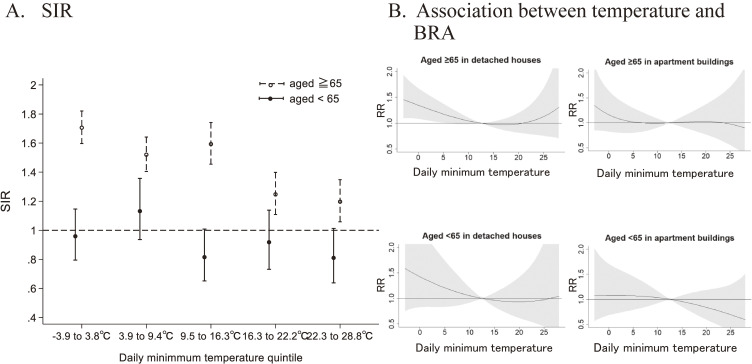
SIR (A) and association between temperature and BRA (B) according to age ≥65 years or <65 years. 4A. The SIR for detached houses compared to apartment buildings by daily minimum temperature quintiles according to aged ≥65 years or <65 years. 4B. The cumulative exposure-response curve of the association between daily minimum temperatures and BRA in detached houses and apartment buildings according to aged ≥65 years or <65 years. Abbreviation: BRA, bathing-related accidents; SIR, age-standardized incident rate; RR, risk ratio

For subgroup analyses, we performed stratified analyses according to the age being ≥65 years or <65 years (Fig. 4). Using SIRs by daily minimum temperature quantile, the ratios of BRA occurrence in detached houses to that in apartment buildings showed that for people aged ≥65 years, the incidence rate in detached houses were significantly higher in all temperature zones, with the hottest quintile at 1.20 (95% CI: 1.06–1.34) and the coldest quintile at 1.71 (95% CI: 1.60–1.82), following the same trend as for the overall population. However, among individuals aged <65 years, the incidence rate between housing types did not show a statistically significant difference across the temperature zones (Fig. 4A). In the analysis using DLNM, the association between cold temperature and the risk of BRAs was not statistically significant for either age group in apartment buildings. In contrast, among people aged ≥65 years living in detached houses, a statistically significant increase in risk was observed as the temperature decreased (Fig. 4B).

We further performed a stratified DLNM analysis based on whether the cause of BRAs was injury or not, as subgroup analyses (Fig. S4). When the cause of BRAs was not injury, the risk increased monotonically with low temperatures in all houses and detached houses. In contrast, when the cause of BRA was injury, the risk was significantly higher at warmer temperatures for all the houses as well as detached houses.

Sensitivity analyses excluding the tenement population to calculate the SIRs showed that, similar to the main results, the incidence rate in detached houses were significantly higher in all temperature zones among people aged ≥65 years, while that between housing types did not show a statistically significant difference across temperature zones among people aged <65 years (Fig. S5). Using the DLNM with a lag time of 21 days instead of 5 days, we found similar conclusions: the risk of BRAs increased significantly with a decrease in temperature in detached houses, but not in apartment buildings (Fig. S6). Exposure based on the daily mean temperature, rather than the daily minimum temperature, did not show an appreciable change in our conclusions (Fig. S7). Furthermore, analyses excluding cases that occurred during the COVID-19 pandemic indicated that the conclusions remained similar (Fig. S8).

## Discussion

In this study, we investigated the associations among housing type, age, ambient temperature, and BRAs in a large Japanese city. Consistent with previous studies [1–9], BRAs occurred more frequently among older age groups and on colder days. The incidence of BRAs in detached houses was higher among older adults and on cold days. Subgroup analysis showed that the risk of BRA due to cold temperatures increased among older adults only in detached houses, but not in apartment buildings; the risks for BRA, older age, and lower temperature were enhanced in detached houses compared to apartment buildings.

Compared with apartment buildings, detached houses are more likely to be wooden, have a larger surface area exposed to the outside air, and are more susceptible to the effects of outside temperatures [2, 10–12]. Consequently, the temperatures in the bathrooms and changing rooms of detached houses are expected to drop on cold days. BRAs, which are particularly common among older adults on cold days, are thought to be associated with increased risk in detached houses. However, for people living in apartment buildings, even among older adults, there was no clear increase in the risk of BRAs on cold days compared to those living in detached houses. Based on the results of the PAF calculations, we estimated that providing detached houses with insulation equivalent to that of apartment buildings could reduce BRA incidence by >10%.

Among developed countries, BRAs among older adults on cold days are particularly common in Japan [2, 31], unlike other countries where they are more prevalent among infants [30]. Among older adults in Japan, the number of deaths from BRAs is roughly twice that from traffic accidents, and this is a public health issue for Japan as the population of older adults grows [2–4]. This is the first epidemiological study to examine BRAs by housing type. We hypothesized that the risk of BRAs is higher in detached houses, wherein indoor temperatures are lower than those in apartment buildings in winter [10–12]. The results of this study supported our hypothesis. Since the World Health Organization (WHO) provided housing and health guidelines [32], there has been increasing attention to low indoor temperatures in winter in Japan associated with various health outcomes other than BRAs, such as infectious [33] and cardiovascular diseases [13, 34, 35]. Further studies from the perspective of housing type may be needed.

In the temperature-specific SIR analyses by temperature quintiles, the SIR for detached houses compared to apartment buildings was higher in even in the middle quintile of temperature (9.5–16.3 °C). A previous study showed that bathing-related mortality rates were higher for people living alone than for those living with their families in Japan [37]. People who live alone take baths in cold bathrooms which have not been recently used by anyone else. Furthermore, if they become ill while bathing, no one can help them, which may result in the need for an ambulance. In Japan, most young people who live alone live in apartment buildings, while more than half of older adults living alone live in detached houses [38].

Notably, the frequent BRAs among older adults in Japan are reportedly due to impaired consciousness in the bathroom [2–4]. The causes of impaired consciousness are unclear, but several have been pointed out [3]: cardiovascular disease owing to increased blood pressure [6–9], a phenomenon called “heat shock” owing to fluctuations in blood pressure [2, 4], and heatstroke-like symptoms owing to immersion in hot water [5]. In the analysis of this study using CCO design controlling for potential confounding by known and unknown factors [23], the risk increased with low temperatures only in detached houses that generally have poor insulation [2, 10–12, 39], suggesting the possibility that the low temperature in the bathroom itself may be causing physiological changes in older adults in the bathroom, leading to loss of consciousness. The age of the building is also related to insulation, with older houses having poorer insulation [40]. Older adults are believed to be more likely than young people to live in older houses, which supports our view that cold induces physiological changes in poorly insulated houses. Therefore, it is necessary to increase the number of autopsies of bathing deaths and conduct further research on BRAs [3, 36]. In Japan, there is an aging population and an existing burden on forensic pathologists and medical examiners. However, considering that these deaths are twice as common as traffic accident deaths [2–4], it may be worth considering concentrating autopsies on bathing deaths may be worth considering.

In the DLNM analyses by cause of BRAs, the risk was increased by low temperatures in detached houses in the analysis using BRAs owing to causes other than injury. This supports our view that low bathroom temperatures can cause physiological changes. Interestingly, heat increases the risk of BRAs due to injury in detached houses. While this is consistent with the fact that detached houses are more sensitive to outside temperatures than apartment buildings [2, 10–12], the robustness and mechanism by which heat increases BRA-associated injuries require further research.

In contrast to previous studies [36, 38, 40–42], this study evaluated the nonlinear relationship between temperature and BRAs based on DLNM [23], which is a well-established framework to describe the nonlinear association between ambient temperature and health outcomes [26, 27, 43]. As a strategy to prevent BRAs, issuing alerts on high-risk days with low temperatures has been proposed. Based on a linear analysis of bathing-related deaths in Kagoshima Prefecture in Japan, Katsuyama et al. proposed to issue alerts when the daily minimum temperature falls below 3.5 °C [36]. In our nonlinear analyses using DLNM, the risk of BRA increased monotonically with decreasing temperature in all participants, and no clear threshold could be determined. Subgroup analysis of older adults (aged ≥65 years) found a significant risk increase at minimum temperatures below 3.9 °C, and an alert below 4 °C may be appropriate.

The main strength of our study is that the data included all emergency ambulance dispatches in Nagoya City. Japan’s ambulance service is funded by taxpayers and is basically free to use, anytime and anywhere, through the emergency number (119) [14]. Therefore, the socioeconomic bias in ambulance use is very small, and we were able to analyze the actual situation throughout the Nagoya City region. However, this study has some limitations. First, this study was conducted in only one city in Japan; therefore, further research in other regions is required to confirm the generalizability of our results. Moreover, there are differences within Japan regarding climate and home insulation. Second, there is the issue of assessing the housing type where BRAs occurred [2]. These assessments are carried out by emergency medical teams. In particular, tenement houses may be misclassified as apartment buildings, potentially underestimating their risk to apartment buildings. In addition, barrier-free shared housing linked to nursing care and medical care, known as “housing for the elderly with services,” is becoming increasingly common in Japan [44]. Even if an older adult living in this housing has a BRA, it is possible that the associated medical staff will respond and emergency services will not. Such cases were not analyzed as BRAs in this study, and the risk of BRAs in apartment buildings may have been underestimated. Third, we did not have detailed information regarding houses and patients, such as indoor (bathroom) temperature, bathroom facilities, family members, lifestyle (e.g., bathing methods), time activity patterns, or behavioral preferences (in response to the environment) [45]. Thus, we were unable to assess whether the impacts of low temperatures differed across these characteristics. Fourth, the study period was April 2016–March 2022; however, the population used to calculate the SIR is representative of the October 2020 Census [16], which fails to consider population changes during that period. Fifth, the results of this study may contain statistical errors. In particular, as there were fewer BRA cases in apartment buildings than in detached houses, it is possible that the nonsignificant increase in BRA risk in apartment buildings was due to large random errors resulting from the relatively small number of cases. There could have also been errors due to multiple age-specific analyses (e.g., a higher incidence in detached houses in the 30–34-years age group). Sixth, the cases in this study were ambulance dispatches only, and did not include those who died during a BRA and were not transported. Therefore, the association between temperature and BRA in the present study is limited. Finally, the study included data on the COVID-19 pandemic. This conclusion did not change even when the sensitivity analysis excluded cases from the COVID-19 period. However, during the COVID-19 pandemic, people’s lives were changed significantly [29, 46], and emergency medical care had been greatly affected.

## Conclusions

We used emergency ambulance dispatch data from Nagoya City in Japan to examine BRAs by housing type. Detached houses had a higher BRA incidence rate than apartment buildings. Being an older adult and experiencing cold days were found to be risk factors for BRAs; however, these risks were more enhanced in detached houses than in apartment buildings. These findings suggest that prevention strategies for BRAs, which are becoming a public health problem in Japan as the population of older adults increases, should focus more on measures involving detached houses.

## Abbreviations

BRA: bathing-related accidents
SIR: age-standardized incidence rate
CI: confidence interval
PAF: population attributable fraction
CCO: case-crossover
RR: risk ratio
DLNM: distributed lag non-linear modeling
df: degree of freedom
QAIC: Quasi Akaike Information Criterion
CPA: cardiopulmonary arrest
WHO: World Health Organization
COVID-19: coronavirus disease 2019

## Supplementary information

**Additional file 1:** **Figure S1.** Population by age group for detached houses and apartment buildings in Nagoya City, Japan. **Figure S2.** Cumulative exposure-response curve of the association between daily minimum temperatures and BRA in all subjects of Nagoya City. **Figure S3.** Cumulative exposure-response curve of the association between daily minimum temperatures and BRA according to aged ≥65 years or <65 years. **Figure S4.** The cumulative exposure-response curve of the association between daily minimum temperatures and BRA in detached houses and apartment buildings according to the case of injury or not. **Figure S5.** The SIR for detached houses compared to apartment buildings by daily minimum temperature quintiles, excluding the tenement houses population. **Figure S6.** The cumulative exposure-response curve of the association between daily minimum temperatures and BRA in detached houses and apartment buildings using the DLNM with a lag time of 21 days. **Figure S7.** The cumulative exposure-response curve of the association between daily minimum temperatures and BRA in detached houses and apartment buildings using exposure based on daily mean temperature. **Figure S8.** The cumulative exposure-response curve of the association between daily minimum temperatures and BRA in detached houses and apartment buildings. **Table S1.** BRA incident rate per 100,000 person-years for detached houses and apartment buildings and incident rate ratio for detached houses compared to apartment buildings. **Table S2.** RR of BRA associated with daily minimum temperature for all subjects, subjects living in detached houses and apartment buildings. **Table S3.** RR of BRA associated with daily minimum temperature for all subjects, subjects living in detached houses and apartment buildings according to aged ≥65 years or <65 years.

## Declarations

### Availability of data and materials

The data that support our findings are available from the Nagoya City Fire Department Emergency Services Division upon reasonable request, but will not be made and are not publicly available.
