# Supplementary material for: Bathing-related accidents requiring ambulance dispatches in relation to age and ambient temperature in Nagoya, Japan: differences between detached houses and apartment buildings
Source: Environ Health Prev Med. 2025 Sep 17;30:72. doi: 10.1265/ehpm.25-00123 (PMC12463714; doi:10.1265/ehpm.25-00123)
Supplement: Supplementary file 1 — Additional file 1: Figure S1. Population by age group for detached houses and apartment buildings in Nagoya City, Japan. Figure S2. Cumulative exposure-response curve of the association between daily minimum temperatures and BRA in all subjects of Nagoya City. Figure S3. Cumulative exposure-response curve of the association between daily minimum temperatures and BRA according to aged ≥65 years or <65 years. Figure S4. The cumulative exposure-response curve of the association between daily minimum temperatures and BRA in detached houses and apartment buildings according to the case of injury or not. Figure S5. The SIR for detached houses compared to apartment buildings by daily minimum temperature quintiles, excluding the tenement houses population. Figure S6. The cumulative exposure-response curve of the association between daily minimum temperatures and BRA in detached houses and apartment buildings using the DLNM with a lag time of 21 days. Figure S7. The cumulative exposure-response curve of the association between daily minimum temperatures and BRA in detached houses and apartment buildings using exposure based on daily mean temperature. Figure S8. The cumulative exposure-response curve of the association between daily minimum temperatures and BRA in detached houses and apartment buildings. Table S1. BRA incident rate per 100,000 person-years for detached houses and apartment buildings and incident rate ratio for detached houses compared to apartment buildings. Table S2. RR of BRA associated with daily minimum temperature for all subjects, subjects living in detached houses and apartment buildings. Table S3. RR of BRA associated with daily minimum temperature for all subjects, subjects living in detached houses and apartment buildings according to aged ≥65 years or <65 years. [file ehpm-30-072-s001.pdf]

**Figure S1. Population by age group for detached houses and apartment buildings in Nagoya City, Japan.**

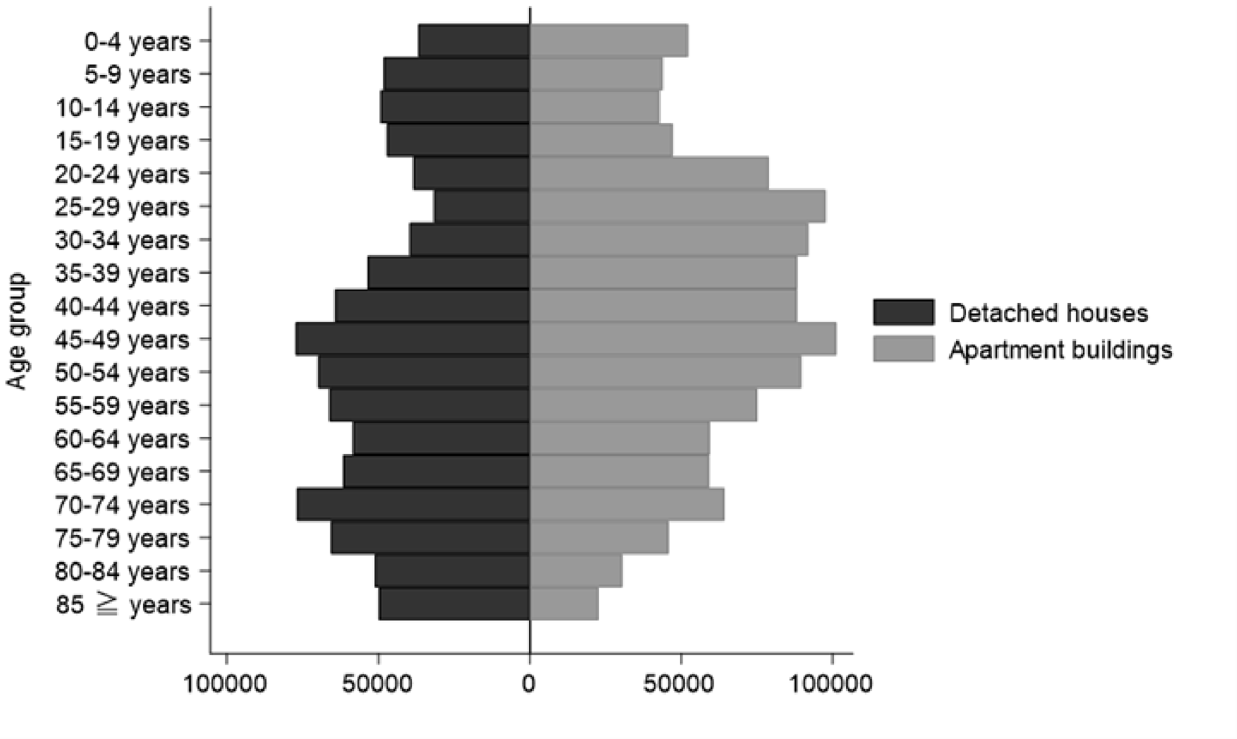

**Figure S2. Cumulative exposure-response curve of the association between daily minimum temperatures and BRA in all subjects of Nagoya City.**

S2A

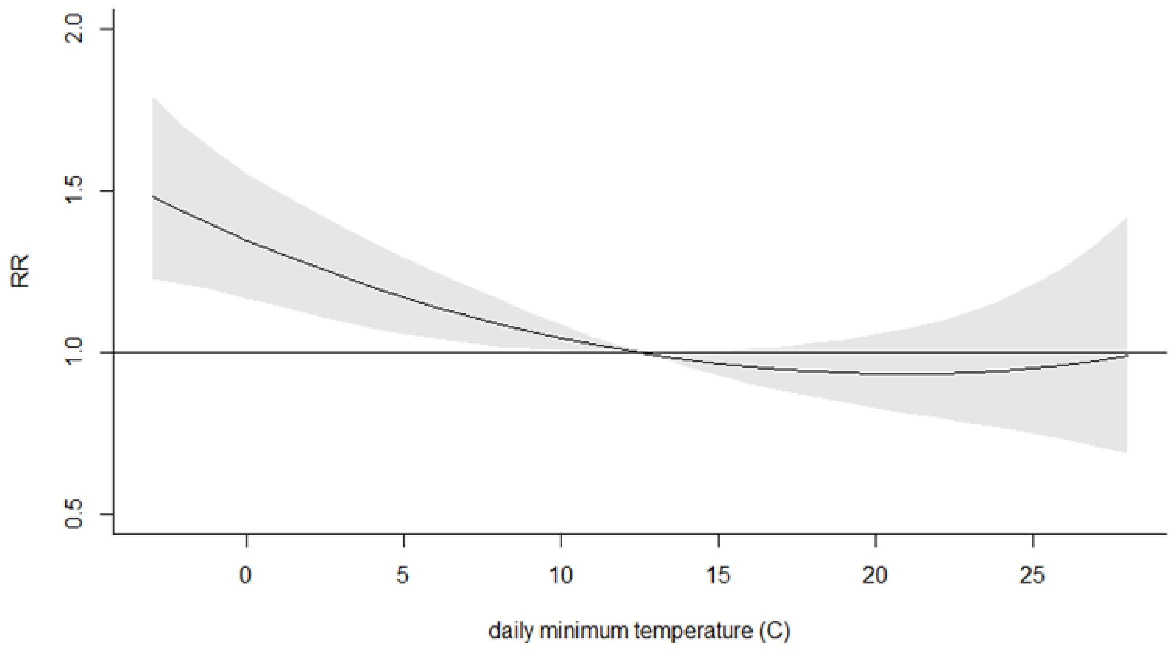


S2B. Three-dimensional graphs of the exposure-response relationship between daily minimum temperature, Lag (0-5 days) and BRA.

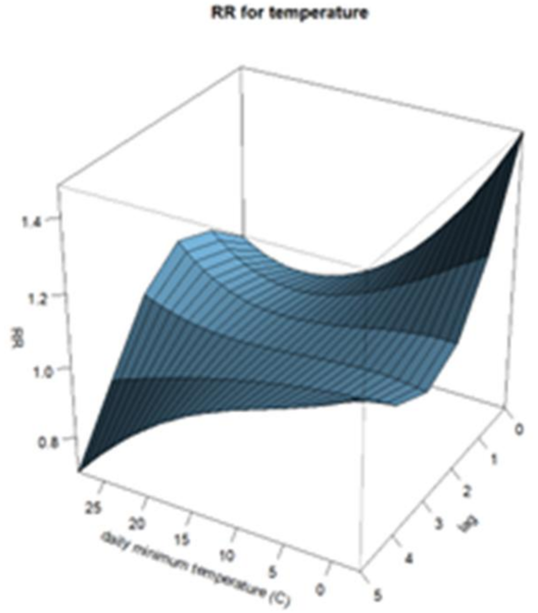


Abbreviation: BRA, bathing-related accidents; RR, risk ratio

**Figure S3. Cumulative exposure-response curve of the association between daily minimum temperatures and BRA according to aged ≥65 years or <65 years.**

S3A. Aged ≥65 years

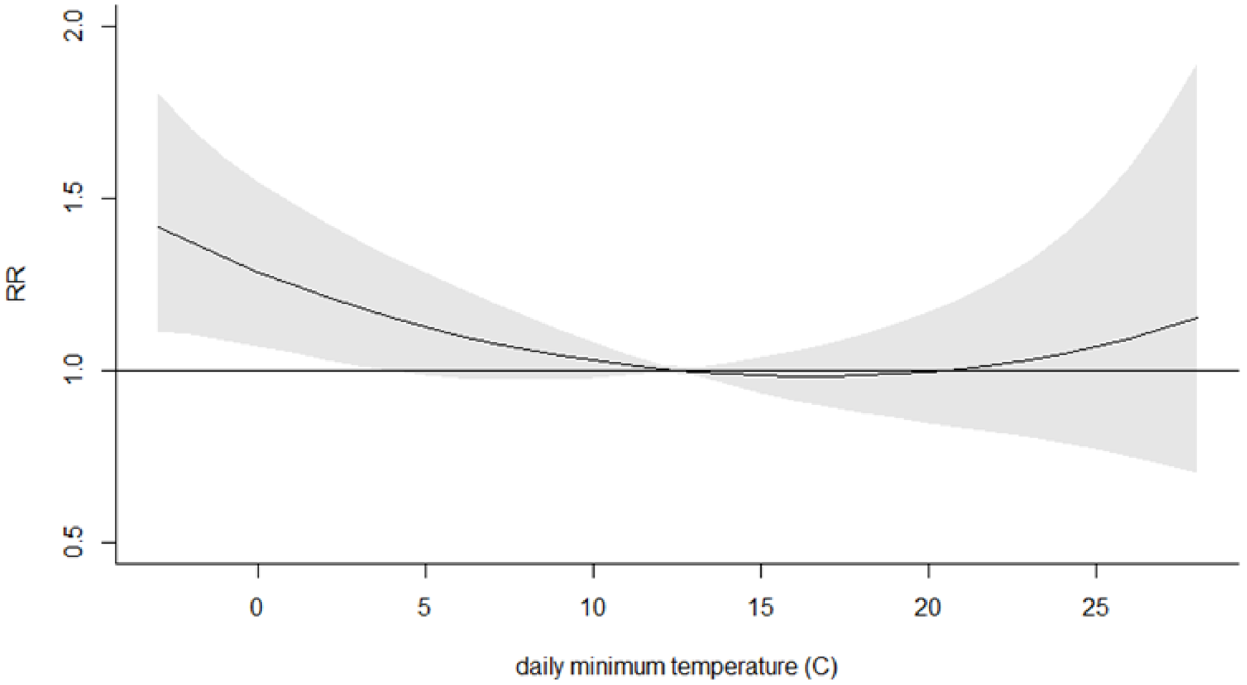


S3B. Aged <65 years

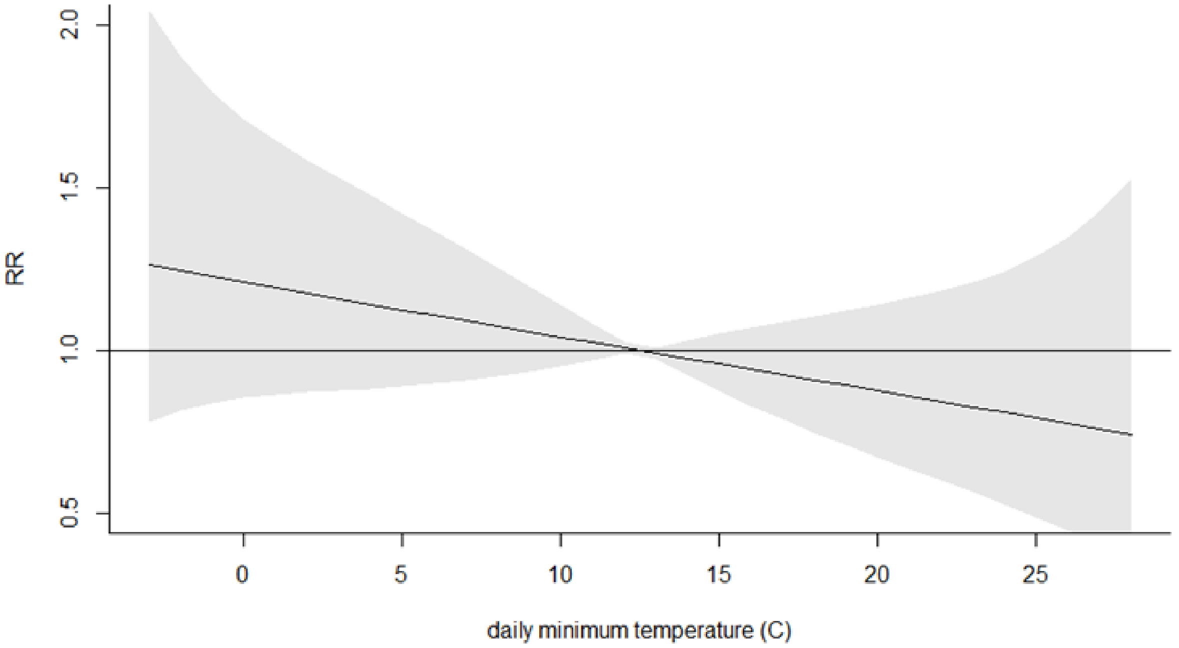


Abbreviation: BRA, bathing-related accidents; RR, risk ratio.

**Figure S4. The cumulative exposure-response curve of the association between daily minimum temperatures and BRA in detached houses and apartment buildings according to the case of injury or not.**

Case of injury in detached houses

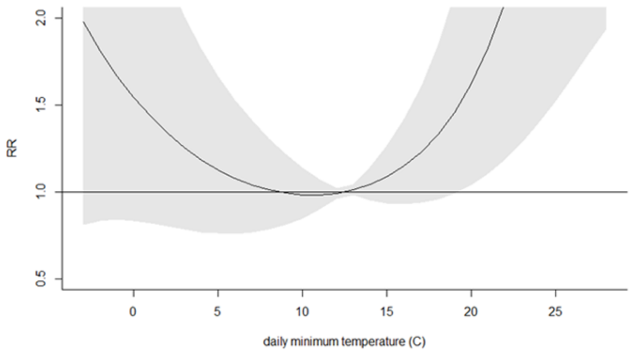


Case of injury in apartment buildings

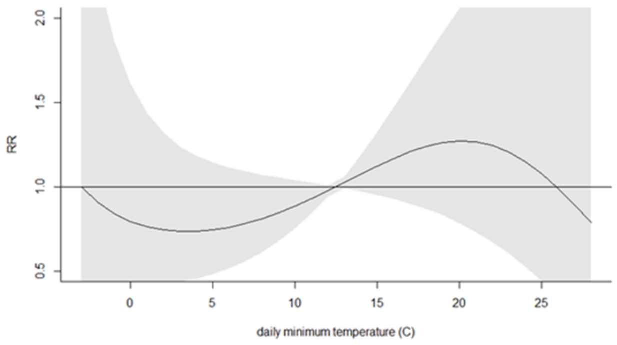


Case of non-injury in detached houses

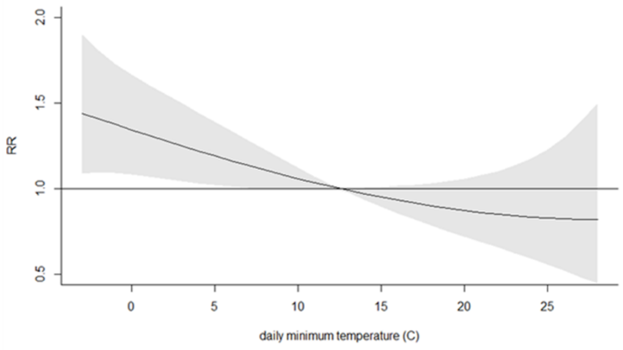


Case of non-injury in apartment buildings

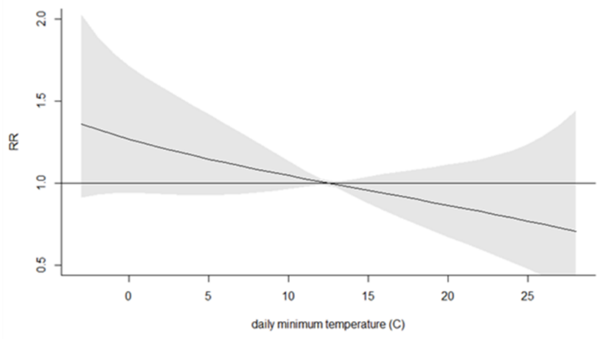


Abbreviation: BRA, bathing-related accidents; RR, risk ratio.

**Figure S5. The SIR for detached houses compared to apartment buildings by daily minimum temperature quintiles, excluding the tenement houses population.**

S5A

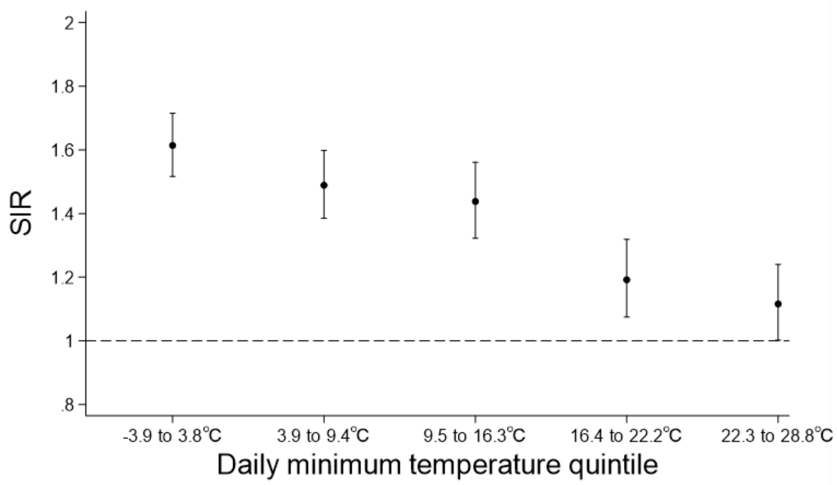


S5B. The SIR for detached houses compared to apartment buildings by daily minimum temperature quintiles according to aged ≥65 years or <65 years.

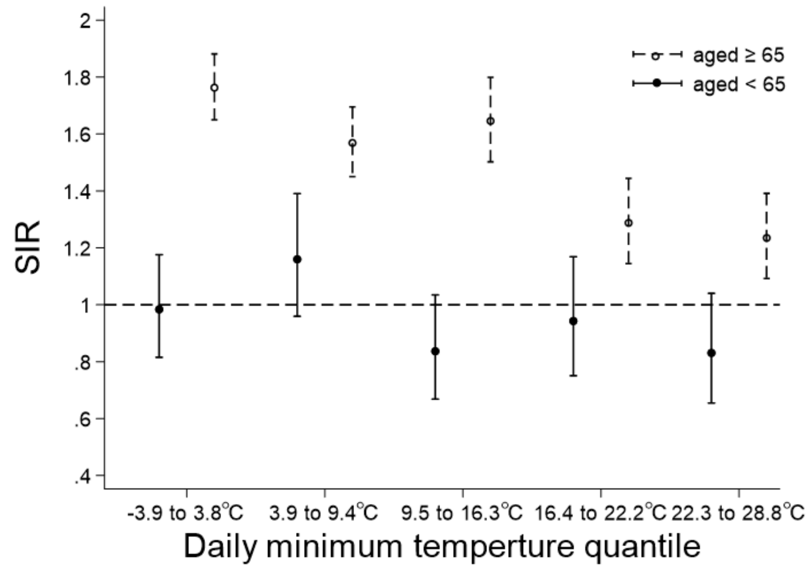


Abbreviation: SIR: age-standardized incidence rate

**Figure S6. The cumulative exposure-response curve of the association between daily minimum temperatures and BRA in detached houses and apartment buildings using the DLNM with a lag time of 21 days.**

S6A. DLNM with a lag time of 21 days in detached houses

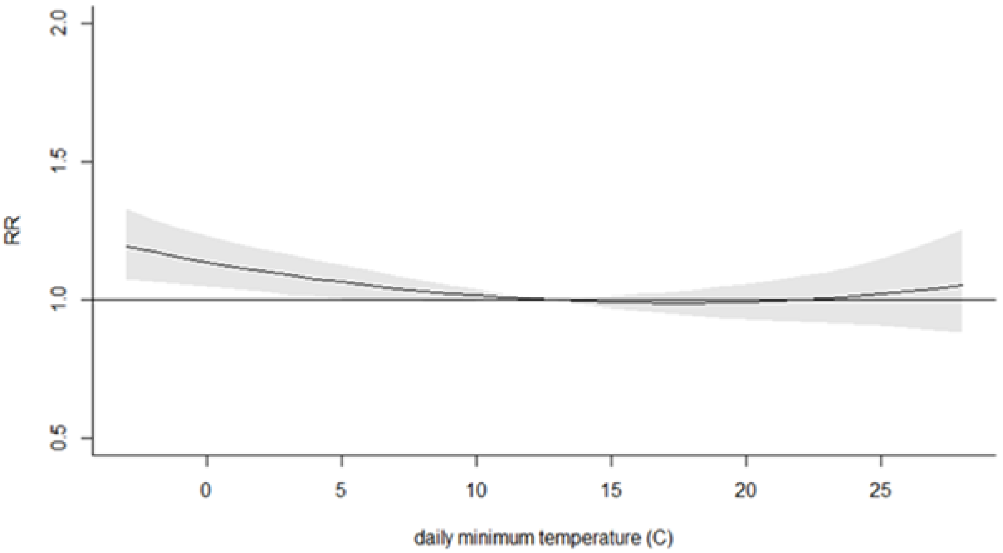


S6B. DLNM with a lag time of 21 days in apartment buildings

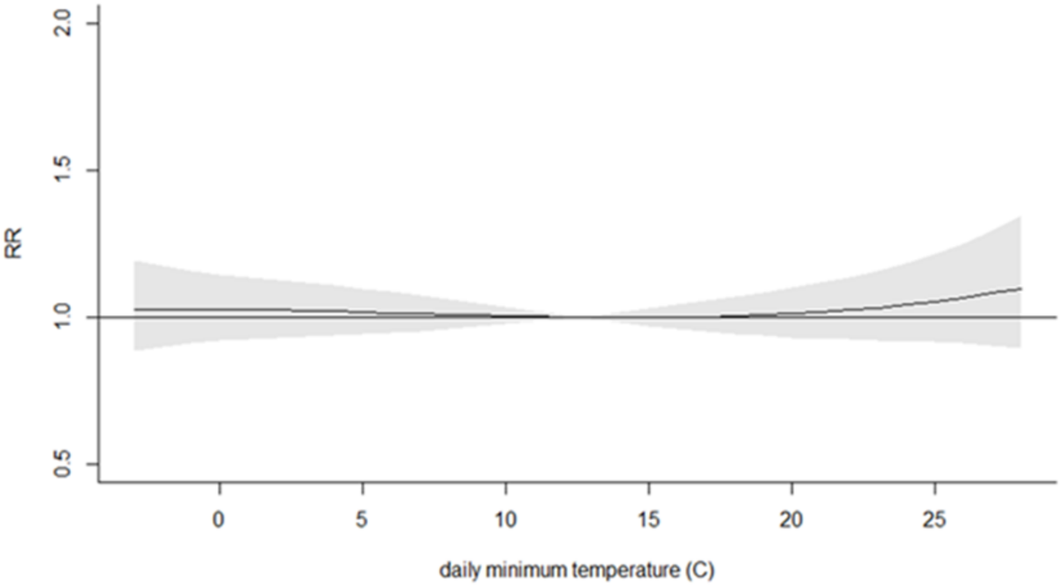


Abbreviation: BRA, bathing-related accidents; DLNM: distributed lag non-linear modeling; RR, risk ratio.

**Figure S7. The cumulative exposure-response curve of the association between daily minimum temperatures and BRA in detached houses and apartment buildings using exposure based on daily mean temperature**

S7A. Detached houses using exposure based on daily mean temperature.

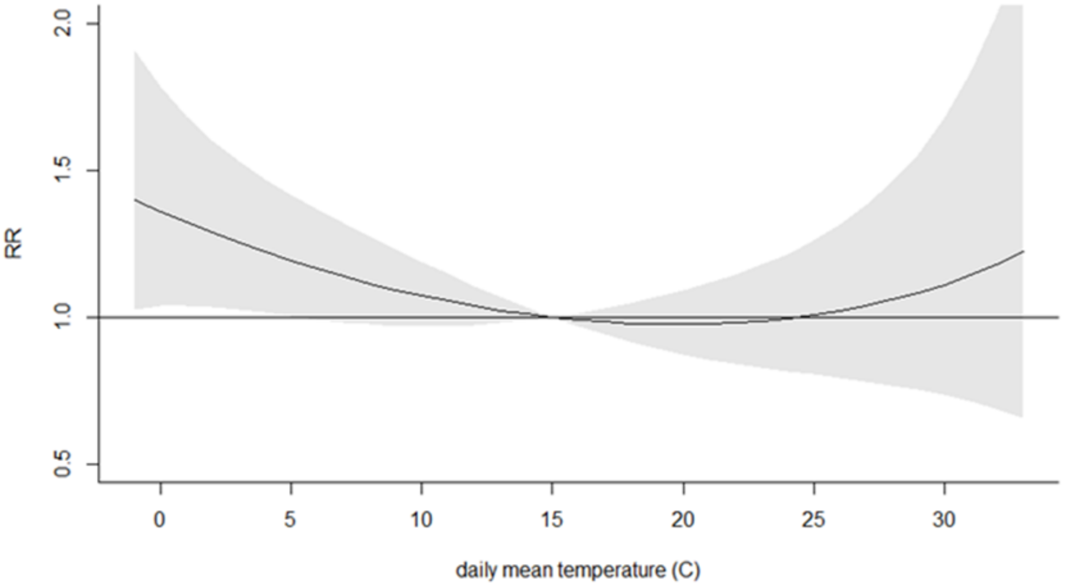


S7B. Apartment buildings using exposure based on daily mean temperature.

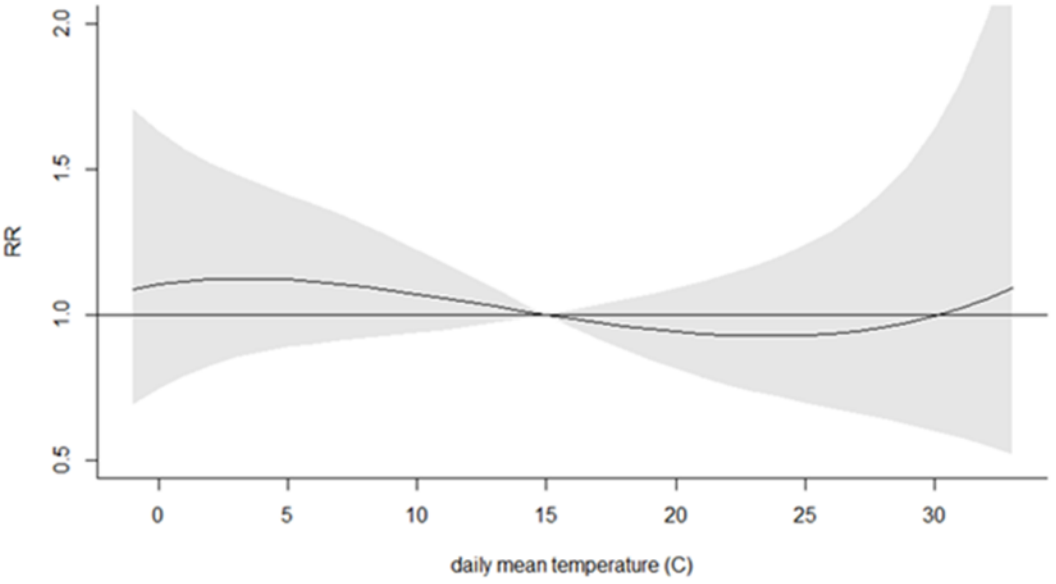


Abbreviation: BRA, bathing-related accidents; RR, risk ratio.

**Figure S8. The cumulative exposure-response curve of the association between daily minimum temperatures and BRA in detached houses and apartment buildings**

Figure S8A. Excluding COVID-19 pandemic period in detached houses

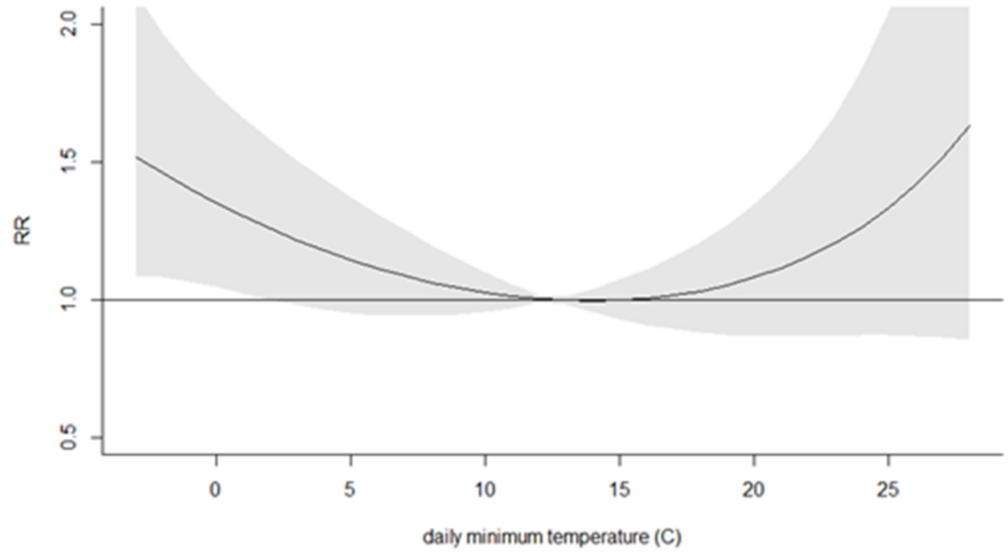


Figure S8B Excluding COVID-19 pandemic period in apartment buildings.

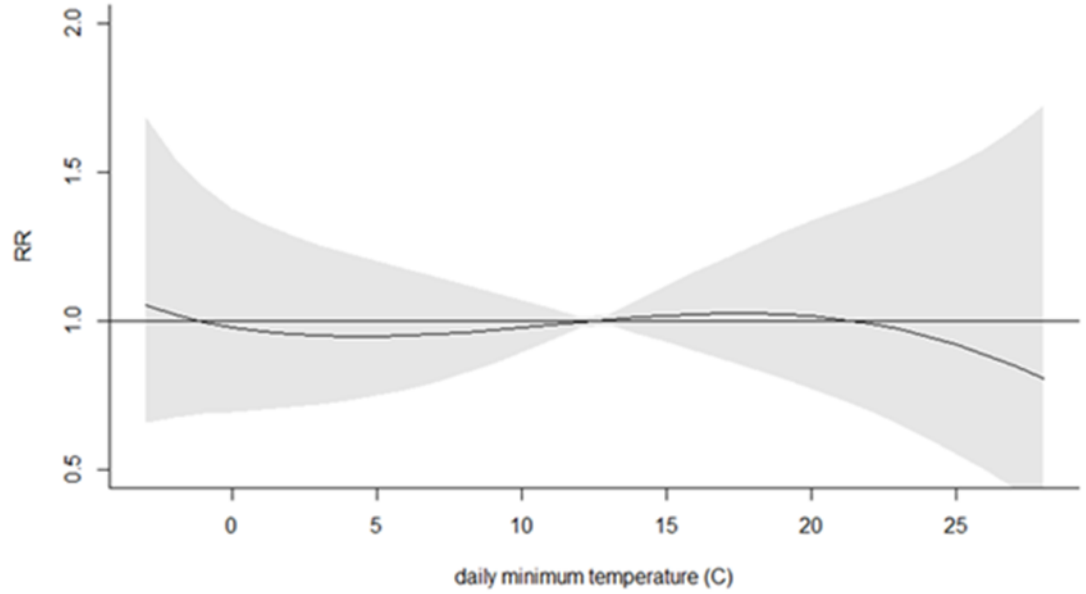


Abbreviation: BRA, bathing-related accidents; COVID-19: coronavirus disease 2019; RR, risk ratio.

**Table S1. BRA incident rate per 100,000 person-years for detached houses and apartment buildings and incident rate ratio for detached houses compared to apartment buildings.**

| Age group | Detached houses | Apartment buildings | Incident rate ratio | 95%CI |
|---|---|---|---|---|
| 0-4 years | 40.2 | 39.9 | 1.01 | 0.76－1.33 |
| 5-9 years | 6.2 | 7.2 | 0.86 | 0.42－1.73 |
| 10-14 years | 9.4 | 11.7 | 0.81 | 0.47－1.40 |
| 15-19 years | 9.2 | 10.6 | 0.86 | 0.49－1.51 |
| 20-24 years | 6.9 | 9.9 | 0.70 | 0.37－1.25 |
| 25-29 years | 5.8 | 8.4 | 0.69 | 0.32－1.35 |
| 30-34 years | 11.3 | 6.2 | 1.83 | 1.07－3.14 |
| 35-39 years | 7.1 | 9.2 | 0.77 | 0.45－1.29 |
| 40-44 years | 6.5 | 6.4 | 1.01 | 0.58－1.74 |
| 45-49 years | 8.0 | 9.1 | 0.88 | 0.56－1.36 |
| 50-54 years | 12.6 | 14.5 | 0.87 | 0.60－1.25 |
| 55-59 years | 14.8 | 13.3 | 1.15 | 0.79－1.68 |
| 60-64 years | 19.6 | 22.4 | 1.17 | 0.84－1.65 |
| 65-69 years | 38.6 | 36.0 | 1.07 | 0.84－1.37 |
| 70-74 years | 71.7 | 41.3 | 1.74 | 1.43－2.11 |
| 75-79 years | 125.2 | 91.0 | 1.38 | 1.18－1.60 |
| 80-84 years | 213.6 | 151.8 | 1.41 | 1.22－1.62 |
| 85 ≧ years | 325.3 | 190.5 | 1.71 | 1.49－1.97 |

Abbreviation: BRA, bathing-related accidents.

**Table S2. RR of BRA associated with daily minimum temperature for all subjects, subjects living in detached houses and apartment buildings.**

| | All subjects | | Detached houses | | Apartment buildings | |
|---|---|---|---|---|---|---|
| Temperature | RR | 95%CI | RR | 95%CI | RR | 95%CI |
| -3 | 1.48 | 1.23-1.79 | 1.47 | 1.13-1.91 | 1.27 | 0.88-1.84 |
| -2 | 1.43 | 1.21-1.70 | 1.43 | 1.13-1.81 | 1.22 | 0.88-1.70 |
| -1 | 1.39 | 1.19-1.62 | 1.39 | 1.12-1.73 | 1.18 | 0.88-1.59 |
| 0 | 1.35 | 1.17-1.55 | 1.36 | 1.11-1.66 | 1.15 | 0.87-1.51 |
| 1 | 1.31 | 1.14-1.50 | 1.32 | 1.09-1.59 | 1.12 | 0.87-1.44 |
| 2 | 1.27 | 1.12-1.44 | 1.28 | 1.07-1.53 | 1.09 | 0.86-1.39 |
| 3 | 1.23 | 1.10-1.39 | 1.25 | 1.05-1.47 | 1.07 | 0.86-1.34 |
| 4 | 1.20 | 1.08-1.34 | 1.21 | 1.04-1.42 | 1.06 | 0.86-1.30 |
| 5 | 1.17 | 1.06-1.30 | 1.18 | 1.02-1.36 | 1.04 | 0.86-1.26 |
| 6 | 1.14 | 1.04-1.25 | 1.15 | 1.01-1.31 | 1.03 | 0.87-1.22 |
| 7 | 1.11 | 1.03-1.21 | 1.12 | 1.00-1.25 | 1.02 | 0.88-1.19 |
| 8 | 1.09 | 1.02-1.16 | 1.09 | 0.99-1.20 | 1.02 | 0.90-1.15 |
| 9 | 1.07 | 1.01-1.12 | 1.07 | 0.99-1.15 | 1.01 | 0.92-1.12 |
| 10 | 1.04 | 1.01-1.09 | 1.05 | 0.99-1.10 | 1.01 | 0.94-1.08 |
| 11 | 1.03 | 1.00-1.05 | 1.03 | 0.99-1.06 | 1.00 | 0.96-1.05 |
| 12 | 1.01 | 1.00-1.02 | 1.01 | 1.00-1.02 | 1.00 | 0.99-1.02 |
| 12.5 | 1 | (Reference) | 1 | (Reference) | 1 | (Reference) |
| 13 | 0.99 | 0.98-1.00 | 0.99 | 0.98-1.00 | 1.00 | 0.98-1.01 |
| 14 | 0.98 | 0.96-1.00 | 0.98 | 0.95-1.01 | 0.99 | 0.95-1.04 |
| 15 | 0.97 | 0.93-1.01 | 0.97 | 0.92-1.03 | 0.99 | 0.92-1.07 |
| 16 | 0.96 | 0.90-1.01 | 0.96 | 0.89-1.04 | 0.98 | 0.89-1.09 |
| 17 | 0.95 | 0.88-1.02 | 0.96 | 0.87-1.07 | 0.98 | 0.85-1.12 |
| 18 | 0.94 | 0.86-1.03 | 0.96 | 0.85-1.09 | 0.97 | 0.82-1.14 |
| 19 | 0.94 | 0.84-1.04 | 0.97 | 0.83-1.12 | 0.96 | 0.79-1.16 |
| 20 | 0.93 | 0.83-1.06 | 0.97 | 0.82-1.16 | 0.94 | 0.76-1.18 |
| 21 | 0.93 | 0.81-1.07 | 0.99 | 0.80-1.21 | 0.93 | 0.72-1.19 |
| 22 | 0.93 | 0.8-1.1 | 1.00 | 0.79-1.27 | 0.91 | 0.68-1.21 |
| 23 | 0.94 | 0.78-1.13 | 1.02 | 0.78-1.34 | 0.89 | 0.64-1.23 |
| 24 | 0.94 | 0.77-1.16 | 1.05 | 0.77-1.43 | 0.86 | 0.60-1.25 |
| 25 | 0.95 | 0.75-1.21 | 1.08 | 0.76-1.54 | 0.83 | 0.55-1.27 |
| 26 | 0.96 | 0.73-1.26 | 1.12 | 0.75-1.69 | 0.80 | 0.50-1.30 |
| 27 | 0.97 | 0.71-1.33 | 1.17 | 0.73-1.88 | 0.77 | 0.45-1.33 |
| 28 | 0.99 | 0.69-1.42 | 1.23 | 0.72-2.12 | 0.74 | 0.39-1.38 |

Abbreviation: RR, risk ratio; BRA, bathing-related accidents.

**Table S3. RR of BRA associated with daily minimum temperature for all subjects, subjects living in detached houses and apartment buildings according to aged ≥65 years or <65 years.**

| | Subject aged ≧65years | | | | | | Subject aged < 65years | | | | | |
|---|---|---|---|---|---|---|---|---|---|---|---|---|
| | All subjects ≧65years | | Detached houses | | Apartment buildings | | All subjects < 65 years | | Detached houses | | Apartment buildings | |
| Temperature | RR | 95%CI | RR | 95%CI | RR | 95%CI | RR | 95%CI | RR | 95%CI | RR | 95%CI |
| -3 | 1.42 | 1.11-1.81 | 1.45 | 1.09-1.93 | 1.35 | 0.85-2.14 | 1.26 | 0.78-2.04 | 1.58 | 0.75-3.32 | 1.07 | 0.57-2.02 |
| -2 | 1.37 | 1.10-1.70 | 1.42 | 1.10-1.83 | 1.28 | 0.85-1.92 | 1.24 | 0.81-1.9 | 1.53 | 0.79-2.94 | 1.07 | 0.61-1.88 |
| -1 | 1.33 | 1.09-1.62 | 1.38 | 1.10-1.75 | 1.21 | 0.84-1.76 | 1.23 | 0.84-1.79 | 1.47 | 0.82-2.65 | 1.08 | 0.65-1.77 |
| 0 | 1.29 | 1.07-1.55 | 1.35 | 1.08-1.68 | 1.16 | 0.82-1.64 | 1.21 | 0.85-1.71 | 1.42 | 0.83-2.44 | 1.08 | 0.68-1.70 |
| 1 | 1.25 | 1.05-1.48 | 1.31 | 1.07-1.61 | 1.12 | 0.81-1.54 | 1.19 | 0.86-1.64 | 1.37 | 0.83-2.26 | 1.08 | 0.71-1.64 |
| 2 | 1.21 | 1.03-1.43 | 1.28 | 1.05-1.55 | 1.08 | 0.80-1.47 | 1.17 | 0.87-1.58 | 1.33 | 0.83-2.11 | 1.08 | 0.73-1.59 |
| 3 | 1.18 | 1.01-1.38 | 1.24 | 1.04-1.49 | 1.06 | 0.79-1.41 | 1.16 | 0.88-1.53 | 1.28 | 0.83-1.98 | 1.08 | 0.75-1.55 |
| 4 | 1.15 | 1.00-1.33 | 1.21 | 1.02-1.43 | 1.04 | 0.79-1.35 | 1.14 | 0.88-1.48 | 1.24 | 0.83-1.86 | 1.08 | 0.77-1.51 |
| 5 | 1.13 | 0.99-1.28 | 1.18 | 1.01-1.38 | 1.02 | 0.80-1.30 | 1.12 | 0.89-1.42 | 1.20 | 0.83-1.74 | 1.07 | 0.79-1.46 |
| 6 | 1.10 | 0.98-1.24 | 1.15 | 1.00-1.32 | 1.01 | 0.81-1.25 | 1.11 | 0.90-1.37 | 1.17 | 0.84-1.63 | 1.07 | 0.81-1.40 |
| 7 | 1.08 | 0.97-1.20 | 1.12 | 0.99-1.26 | 1.00 | 0.82-1.21 | 1.09 | 0.91-1.31 | 1.14 | 0.85-1.52 | 1.06 | 0.83-1.35 |
| 8 | 1.06 | 0.97-1.16 | 1.09 | 0.98-1.21 | 0.99 | 0.85-1.17 | 1.07 | 0.92-1.25 | 1.11 | 0.87-1.41 | 1.05 | 0.86-1.29 |
| 9 | 1.04 | 0.97-1.12 | 1.07 | 0.98-1.16 | 0.99 | 0.87-1.13 | 1.06 | 0.94-1.20 | 1.08 | 0.89-1.31 | 1.04 | 0.89-1.23 |
| 10 | 1.03 | 0.98-1.08 | 1.04 | 0.98-1.11 | 0.99 | 0.90-1.09 | 1.04 | 0.95-1.14 | 1.05 | 0.92-1.21 | 1.03 | 0.92-1.16 |
| 11 | 1.02 | 0.98-1.05 | 1.02 | 0.99-1.06 | 0.99 | 0.94-1.05 | 1.02 | 0.97-1.08 | 1.03 | 0.95-1.12 | 1.02 | 0.95-1.10 |
| 12 | 1.00 | 0.99-1.01 | 1.01 | 1.00-1.02 | 1.00 | 0.98-1.02 | 1.01 | 0.99-1.03 | 1.01 | 0.98-1.04 | 1.01 | 0.98-1.03 |
| 12.5 | 1 | (Reference) | 1 | (Reference) | 1 | (Reference) | 1 | (Reference) | 1 | (Reference) | 1 | (Reference) |
| 13 | 1.00 | 0.99-1.01 | 0.99 | 0.98-1.01 | 1.00 | 0.98-1.02 | 0.99 | 0.97-1.01 | 0.99 | 0.96-1.02 | 0.99 | 0.97-1.02 |
| 14 | 0.99 | 0.96-1.02 | 0.98 | 0.95-1.02 | 1.01 | 0.95-1.07 | 0.98 | 0.92-1.03 | 0.98 | 0.89-1.06 | 0.97 | 0.91-1.05 |
| 15 | 0.99 | 0.94-1.04 | 0.97 | 0.92-1.04 | 1.01 | 0.92-1.12 | 0.96 | 0.88-1.05 | 0.96 | 0.83-1.11 | 0.96 | 0.85-1.08 |
| 16 | 0.98 | 0.91-1.06 | 0.97 | 0.89-1.06 | 1.02 | 0.89-1.17 | 0.94 | 0.83-1.07 | 0.95 | 0.78-1.16 | 0.94 | 0.79-1.10 |
| 17 | 0.98 | 0.89-1.08 | 0.97 | 0.87-1.08 | 1.02 | 0.86-1.22 | 0.93 | 0.79-1.09 | 0.94 | 0.73-1.22 | 0.91 | 0.74-1.13 |
| 18 | 0.98 | 0.88-1.11 | 0.97 | 0.84-1.11 | 1.03 | 0.83-1.27 | 0.91 | 0.75-1.11 | 0.94 | 0.69-1.28 | 0.89 | 0.69-1.15 |
| 19 | 0.99 | 0.86-1.14 | 0.98 | 0.83-1.15 | 1.03 | 0.80-1.32 | 0.89 | 0.71-1.12 | 0.93 | 0.65-1.34 | 0.87 | 0.64-1.17 |
| 20 | 1.00 | 0.85-1.17 | 0.99 | 0.81-1.2 | 1.03 | 0.77-1.37 | 0.88 | 0.67-1.14 | 0.93 | 0.62-1.42 | 0.84 | 0.59-1.19 |
| 21 | 1.01 | 0.83-1.21 | 1.00 | 0.80-1.26 | 1.02 | 0.73-1.43 | 0.86 | 0.64-1.16 | 0.94 | 0.58-1.50 | 0.81 | 0.55-1.20 |
| 22 | 1.02 | 0.82-1.26 | 1.02 | 0.79-1.33 | 1.02 | 0.70-1.49 | 0.84 | 0.6-1.18 | 0.94 | 0.55-1.60 | 0.78 | 0.50-1.22 |
| 23 | 1.03 | 0.81-1.32 | 1.05 | 0.78-1.42 | 1.01 | 0.65-1.56 | 0.83 | 0.56-1.21 | 0.95 | 0.52-1.73 | 0.75 | 0.46-1.23 |
| 24 | 1.05 | 0.79-1.39 | 1.09 | 0.77-1.54 | 1.00 | 0.61-1.63 | 0.81 | 0.53-1.24 | 0.96 | 0.49-1.90 | 0.72 | 0.41-1.26 |
| 25 | 1.07 | 0.77-1.48 | 1.13 | 0.75-1.68 | 0.98 | 0.56-1.72 | 0.79 | 0.49-1.29 | 0.97 | 0.45-2.11 | 0.69 | 0.37-1.29 |
| 26 | 1.09 | 0.75-1.59 | 1.18 | 0.74-1.87 | 0.96 | 0.50-1.83 | 0.78 | 0.45-1.35 | 0.99 | 0.41-2.39 | 0.66 | 0.32-1.34 |
| 27 | 1.12 | 0.73-1.73 | 1.24 | 0.73-2.11 | 0.93 | 0.44-1.95 | 0.76 | 0.40-1.43 | 1.02 | 0.37-2.78 | 0.63 | 0.28-1.41 |
| 28 | 1.15 | 0.70-1.89 | 1.31 | 0.71-2.42 | 0.90 | 0.38-2.11 | 0.74 | 0.36-1.53 | 1.04 | 0.33-3.32 | 0.59 | 0.23-1.51 |

Abbreviation: RR, risk ratio; BRA, bath-ingrelated accidents.
